# The genetic profiles and maternal origin of local sheep breeds on Java Island (Indonesia) based on complete mitochondrial DNA D-loop sequences

**DOI:** 10.14202/vetworld.2020.2625-2634

**Published:** 2020-12-11

**Authors:** Alek Ibrahim, I Gede Suparta Budisatria, Rini Widayanti, Wayan Tunas Artama

**Affiliations:** 1 Veterinary Science Postgraduate Study Program, Faculty of Veterinary Medicine, Universitas Gadjah Mada, Yogyakarta, Indonesia; 2 Department of Animal Production, Faculty of Animal Science, Universitas Gadjah Mada, Yogyakarta, Indonesia; 3 Department of Biochemistry and Molecular Biology, Faculty of Veterinary Medicine, Universitas Gadjah Mada, Yogyakarta, Indonesia

**Keywords:** control region, genetic variation, indigenous sheep, Indonesian sheep, non-coding region, phylogenetic

## Abstract

**Background and Aim:**

Java Island is one of the islands in Indonesia which has local sheep breeds with specific characteristics and native development geography in certain regions. This study aimed to determine the genetic profiles and maternal origin of six local sheep breeds on Java Island.

**Materials and Methods:**

This study was conducted by identifying the profiles of complete mitochondrial DNA (mtDNA) displacement loop (D-loop) region sequences on a total of 22 individual in six local sheep breeds on Java Island, including Javanese thin-tailed (JTT), Javanese Fat-Tailed (JFT), Batur (BTR), Wonosobo (WSB), Garut (GRT), and Priangan (PRG) sheep. The D-loop region was amplified using specific primers, and the polymerase chain reaction (PCR) was performed. The PCR products were purified and sequenced.

**Results:**

The mtDNA D-loop analysis identified 21 haplotypes in the analyzed 22 animals with 123 polymorphic sites (V) consisting of 60 singleton variable sites (S) and 63 parsimony informative sites (P). Within all breeds tested, the haplotype diversity, the average number of pairwise differences (K), and nucleotide diversity (Pi) were 0.99567, 25.36364, and 0.02153, respectively. The genetic distance (D) within groups and between groups was 0.001-0.006 and 0.004-0.036, respectively. The phylogeny resulted in the presence of two haplogroups (Hap), which are 5 Hap A and 16 Hap B. All JTT, JFT, BTR, and WSB breeds were in the same cluster in Hap B, whereas GRT and PRG breeds were in clusters in both Hap A and Hap B.

**Conclusion:**

The high genetic diversity in six local sheep breeds on Java Island suggests that they originated from different genetic sources. JTT sheep have closer genetic relationships to JFT, BTR, and WSB sheep, and they are close to European sheep, whereas GRT sheep have closer genetic relationships to PRG sheep. Both are closer to Asian sheep than to European sheep.

## Introduction

Indonesia is an archipelago country that has specific characteristics in each Island and a variety of natural and human resources. Java Island is one of the islands in Indonesia, which consists of four provinces (Banten, West Java, Central Java, and East Java) and two special regions (Jakarta and Yogyakarta). In general, these regions have similar characteristics, but in certain regions, they have unique characteristics regarding geographic features, natural resources, social, and cultural background. The character of the region and different natural resources dictate the type a socio-cultural community that will be established, at least with respect to farming activities. Farming activities adjust to the conditions of the area, including the selection of livestock that is suitable for rising. Sheep are one of the many livestock raised by farmers on Java Island that have a notable value in society [1,2]. Sheep play key roles in providing food, clothing, the raw material for traditional housing [3], agriculture, economy [4,5], religious festivities, and cultural/traditional festivals [6-8]. The local sheep are sheep breeds raised by local people in particular area and proven to be adaptable in that area [9]. At present, eight local Indonesian sheep breeds have been established by the Ministry of Agriculture of the Republic of Indonesia, four of which were developed in the original area on Java Island, namely, Garut (GRT), Batur (BTR), Wonosobo (WSB), and Priangan (PRG) sheep. Each breed has phenotypic characteristics that are easily recognized, but genetically many things have not been studied. Local Indonesian sheep that are developing at this time might be the result of a cross between thin-tailed sheep or fat-tailed sheep [10,11]. These sheep types have already been developed by the community with other various breeds that were imported from abroad to form new sheep breeds in the development area. However, the genetic origin of local sheep is still not known with certainty.

One method to determine genetic diversity, relationships and origins of local Indonesian sheep are to examine the mitochondrial DNA (mtDNA) profile. The mtDNA is an important genetic study tool in population and systematic molecular genetics because it has many copies with a rapid evolutionary rate and is inherited [12]. One part of mtDNA that can be observed is the displacement loop (D-loop) region or control region. The D-loop is a segment of mtDNA that acts as the initial intermediary for replication and is more variable than are other mtDNA regions [3]. Since the D-loop exhibits extraordinary levels of variation within species, it can be used to track geographical patterns of diversity and evolution (phylogeographic), distribution, gene flow, maternal origin, demographic expansion, genetic drift, population structures, and hybridization [13-15]. The use of the mtDNA D-loop sequence to determine the origin of sheep has been conducted in several wild sheep and domesticated sheep from Asia, Europe, and New Zealand [16,17]. However, no in-depth study of the genetic profile and origin of all local sheep breeds on Java Island according to breed have ever been determined based on the complete D-loop sequence.

This study aimed to determine the complete mtDNA D-loop sequences of six local sheep breeds on Java Island, Indonesia. The results of this study can be used to determine genetic diversity, genetic distance, and relationships between local sheep breeds and other species of sheep on Java Island. Furthermore, the results can be added to databases and used to complement previous studies on genetic diversity and relationships of local Indonesian sheep, especially on Java Island.

## Materials and Methods

### Ethical approval

This study was approved by the Animal Care and Use Committee of the Faculty of Veterinary Medicine, Universitas Gadjah Mada, with the ethical clearance number 002/EC-FKH/Int/2019, and the National Political and Unity of Yogyakarta Special District with the approval number 074/1850/Kesbangpol/2019.

### Sample collection

**Figure-1:**
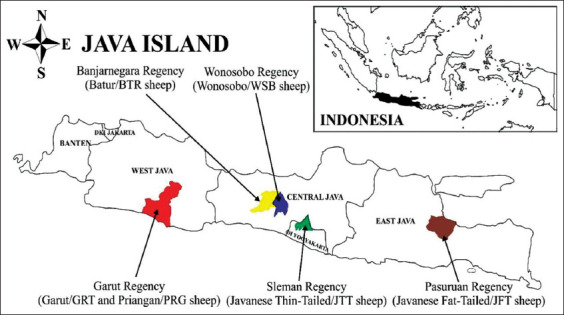
Sampling sites local sheep on Java Island, Indonesia.

This study was conducted using blood samples from 22 individual sheep in six populations of local sheep breeds on Java Island and is presented in Figure-1. Sheep were sampled by a purposive sampling method, namely, by determining the local district sheep population center in the regency, and then the specified districts and villages. This study was conducted from April 2019 to February 2020. Blood was drawn using a 3 mL syringe through the jugular vein that had been previously cleaned with alcohol. The blood was then collected in vacutainer tubes with anticoagulant (ethylenediaminetetraacetic acid), and then was stored in a cooler box containing an ice pack and transported to the laboratory for further analysis.

### Molecular techniques

The DNA was extracted based on the manufacturer’s standard protocol using PureLink™ Genomic DNA Mini Kits (Invitrogen, USA). The mtDNA D-loop was amplified directly from the genomic DNA by polymerase chain reaction (PCR). The primer was designed using the Primer3 online version 4.1.0 program (http://primer3.UT.ee//) [18] based on the data from the mitochondria genome of *Ovis aries* (GenBank accession number: AF010406.1). The mtDNA D-loop region primer sequences were Alek-DLF: 5’-GAAGAAGCTATAGCCCCACT-3’ and Alek-DLR: 5’-GATTCGAAGGGCGTTACT-3’ that generated 1397 bp of the PCR product. The PCR reaction consisted of 4 μL of DNA template, 25 μL of KAPA2G Fast Ready Mix+Dye (Kapa Biosystems Ltd.), 2 μL of forward primer, 2 μL of reverse primer, and 17 of ddH_2_O. The PCR amplification was conducted using Cleaver GTC96S (Cleaver Scientific Ltd.) according to the program: 6-min of pre-denaturation at 94°C, followed by 35 cycles, each consisting denaturation at 94°C for 30-s, primers annealing at 47°C for 40-s, extension at 72°C for 90-s, then ending with a final extension at 72°C for 5-min, and storage at 4°C. The PCR product was visualized using 1.5% agarose gel, and electrophoresis was run at 100 mV for 30-min. The result of amplification could be seen on the UV illuminator. The purified PCR products were sequenced by 1^st^ BASE-Asia, Malaysia.

### Statistical analysis

The product length of the D-loop region sequences amplification for each individual is 1800 bp and was analyzed using the molecular evolutionary genetics analysis (MEGA) version 7.0 software (Pennsylvania State University, USA) [19]. The D-loop region sequences of local sheep were aligned using the Clustal W program [20]. The analysis of genetic profiles was determined by the difference in the nucleotide sequence of the D-loop region. Genetic distance was analyzed using the Kimura 2-parameter method [21]. Genetic diversity and haplotype diversity (HD) were analyzed using DNA Sequence Polymorphism version 6 software (Universitat de Barcelona, Spain) [22].

Reference mtDNA D-loop region sequences of wild and domestic sheep of known haplogroup (Hap) types [23] were downloaded from GenBank and used as comparators of local sheep breeds on Java Island to look up phylogenetic trees and assume their origin (Table-1). The phylogenetic tree was constructed based on the D-loop region sequences using the neighbor-joining method [24] with bootstrap test 1000× replication [25] and Kimura 2-parameter method [21] in the MEGA program [19].

**Table-1:** References of domestic and wild sheep.

Group membership	GenBank accession number
*Ovis aries*	
Haplogroup A	HM236174, HM236175
Haplogroup B	HM236176, HM236177
Haplogroup C	HM236178, HM236179
Haplogroup D	HM236180, HM236181
Haplogroup E	HM236182, HM236183
*Ovis musimon*	HM236184
*Ovis vignei*	HM236187
*Ovis ammon*	HM236188
*Ovis orientalis*	KF312238
*Ovis canadensis*	MH094035
*Ovis nivicola*	MH779626
*Ovis dalli*	MH779627

## Results

### Genetic diversity and HD

All local sheep breeds on Java Island have D-loop sequences of 1180 bp except for one sample (GRT2) that has a length of 1181 bp. The length of this sequence, as the long sequence of the D-loop on *Ovis aries* (AF010406.1), was equal to 1180 bp. However, differences were found in the sequence at 908 where C nucleotide insertion and deletion in the sequence at 1037 except in the GRT2 sample, which still has a T nucleotide at the site.

**Figure-2:**
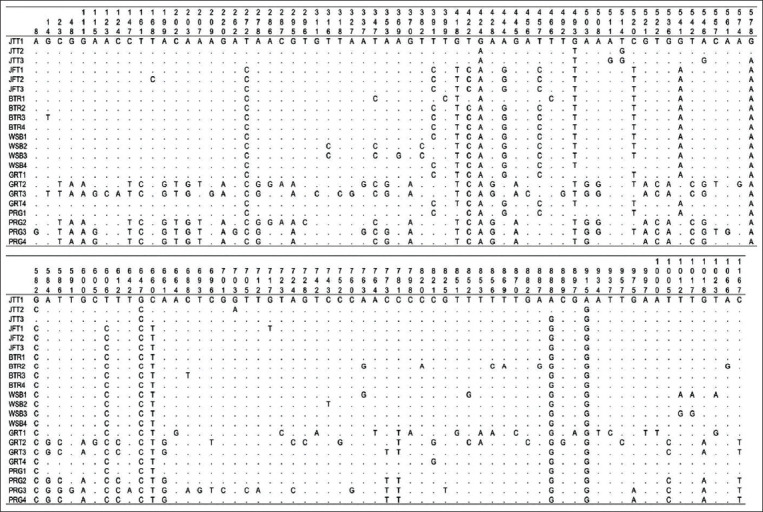
Polymorphic sites of mitochondrial DNA D-loop in the local sheep breeds on Java Island.

The alignment of mtDNA D-loop nucleotide sequences of local sheep breeds on Java Island enabled polymorphic site data to be obtained and is presented in Figure-2. The average percentage of T, A, C, and G nucleotides were 29.5%, 31.1%, 22.9%, and 14.4%, respectively. The percentage of A+T and C+G nucleotide pairs was 62.6% and 37.4%, respectively. Based on the aligned D-loop mtDNA nucleotide sequence (Figure-2), specific nucleotide substitutions were obtained that might represent the genetic markers in each breed. The Javanese thin-tailed (JTT) has four unique polymorphic sites, namely, at sites 272, 422, 606, and 660. The unique polymorphic has not been found in the other breed that can distinguish it from other breeds.

The parameters of genetic diversity and HD of the mtDNA D-loop region in six subpopulations of local sheep breeds on Java Island are as shown in Table-2. The sequencing of the six local sheep breeds on Java Island generated sequences of the D-loop region throughout 1180 bp and 1 indel (insertion or deletion) (1180+1:1181). It showed that there were 123 polymorphic sites (V) (Figure-2), which consisted of 63 parsimony informative sites (P) and 60 singleton variable sites (S), and 130 of the total number of mutations. From 22 samples, there were 21 haplotypes (Hap) with 20 unique haplotypes and 1 shared haplotype (Hap6: JFT3 and BTR4). The average HD, the average number of pairwise differences (K), and nucleotide diversity (Pi) of the complete mtDNA D-loop in six local sheep breeds of Java Island were 0.99567, 25.36364, and 0.02153, respectively.

**Table-2:** Genetic diversity and haplotype diversity of local sheep breeds on Java Island based on complete mtDNA D-Loop sequence.

Breeds	n	V	S	P	Indel	nHap	HapA	HapB	HD	K	Pi
All	22	123	60	63	1	21	5	16	0.99567	25.36364	0.02153
JTT	3	11	11	0	0	3	0	3	1.00000	7.33333	0.00623
JFT	3	2	2	0	0	3	0	3	1.00000	1.33333	0.00113
BTR	4	15	15	0	0	4	0	4	1.00000	7.50000	0.00637
WSB	4	12	8	4	0	4	0	4	1.00000	7.33333	0.00623
GRT	4	80	43	37	1	4	2	2	1.00000	45.16667	0.03834
PRG	4	62	57	5	0	4	3	1	1.00000	31.66667	0.02688

JTT=Javanese thin-tailed sheep, JFT=Javanese fat-tailed sheep, BTR=Batur sheep, WSB=Wonosobo sheep, GRT=Garut sheep, PRG=Priangan sheep. n=Number of samples, V=Polymorphic sites, S=Singleton variable sites, P=Parsimony informative sites, Indel=Insertion and deletion. nHap=Number of haplotypes, HapA=Haplogroup A, HapB=Haplogroup B, HD=Haplotype diversity, K=Average number of pairwise different, Pi=Nucleotide diversity

### Genetic distance

The value of genetic distance within and between groups is presented in Table-3. The genetic distance value in local sheep groups on Java Island is 0.001-0.040, with the lowest in JTT sheep and the highest in GRT sheep. The genetic distance values between groups of local sheep breeds range between 0.004 and 0.036. The lowest genetic distance is between Javanese fat-tailed (JFT) and BTR or WSB, whereas the highest is between JTT and PRG. The JFT, BTR, and WSB populations have a high genetic distance value with GRT and PRG.

**Table-3:** Genetic distance values within and between groups of local sheep breeds on Java Island based on complete mtDNA D-Loop sequence.

Population	JTT	JFT	BTR	WSB	GRT	PRG
JTT	**0.006**					
JFT	0.013	**0.001**				
BTR	0.014	0.004	**0.006**			
WSB	0.015	0.004	0.007	**0.006**		
GRT	0.034	0.026	0.028	0.029	**0.040**	
PRG	0.036	0.031	0.032	0.032	0.030	**0.028**

JTT=Javanese thin-tailed sheep, JFT=Javanese Fat-Tailed sheep, BTR=Batur sheep, WSB=Wonosobo sheep, GRT=Garut sheep, PRG=Priangan sheep. Genetic distance values between groups are shown in normal and within groups are shown in bold

### Phylogenetic relationship

**Figure-3:**
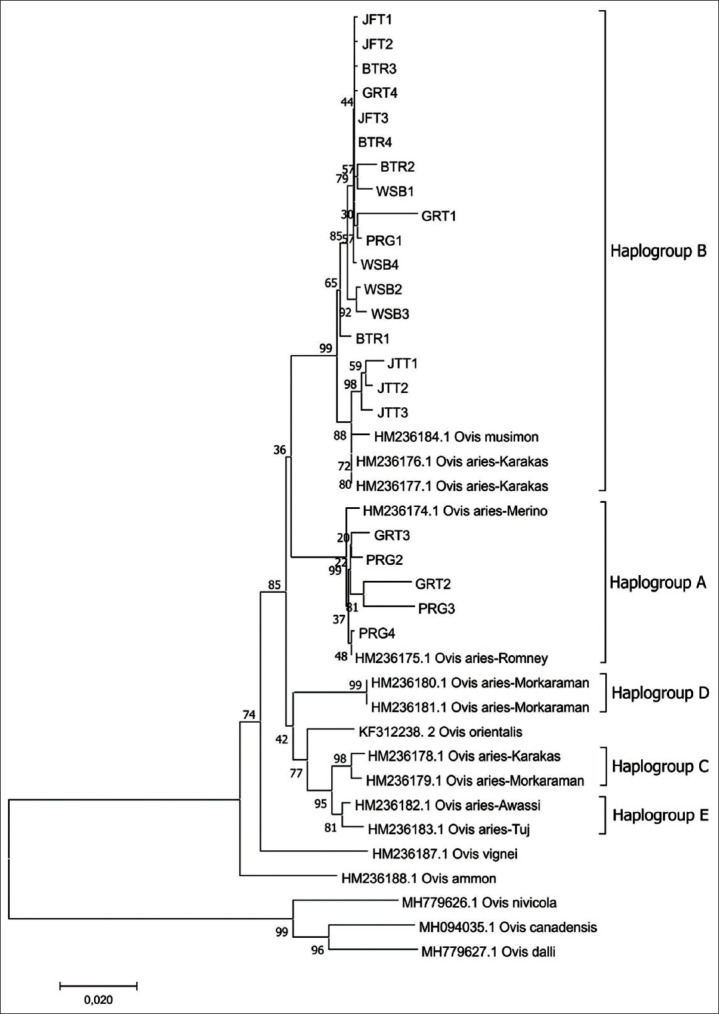
Phylogenetic tree of six local sheep breeds on Java Island compared with other species of sheep.

The phylogenetic tree of 21 haplotypes in six subpopulations of local sheep breeds on Java Island and mtDNA D-loop sequences of wild sheep and domestic sheep categorized by Hap from GenBank are presented in Figure-3. The phylogenetic analysis showed that local sheep breeds on Java Island are divided into two Hap, namely, Hap A and B. All JTT, JFT, BTR, and WSB sheep are categorized into Hap B.

## Discussion

### Genetic diversity and genetic distance of local sheep breeds on Java Island, Indonesia

The sequence length, in this study, is the same as the length of the D-loop sequence in *O. aries* (AF010406.1), which is 1800 bp, but there is a difference at the 908 site where the C nucleotide is inserted. A deletion occurs at the 1037 site except for GTR2 samples (Figure-1), which has 1 indel (Table-2), but still has a T nucleotide on the site. The sequence length and characteristics of the T nucleotide at the 1037 site on GRT2 are similar to those of the *Ovis orientalis* (KF312238). The nucleotide composition of six local sheep on Java in this study was almost similar to Tibetan sheep [26,27], Small-Tailed Hulun Buir sheep, Shandong Large-Tailed sheep, Altay sheep [28], and Alpine Merino sheep [29], with the A+T pairs were substantially more common than the G+C pairs.

Genetic diversity, besides reflecting differences in traits between individuals, also reflects small differences in nucleotides [15]. Genetic diversity (Pi) values ranged from 0.001 to 0.01 and defined three categories, that is, a high category that ranged from 0.008 to 0.01, a medium category that ranged from 0.005 to 0.007, and a low category that ranged from 0.001 to 0.004 [30]. The analysis of mtDNA D-loop sequences in local sheep breeds on Java Island showed a high genetic diversity between breeds (0.02153) and within breeds (0.00623-0.03834), except for JFT (0.00113), which belonged to the medium category (Table-2). The values obtained in this study are similar to the genetic diversity within breeds of ten Iranian indigenous breeds (0.00871-0.04762) [31], lower than five Egyptian sheep breeds (0.01433-0.03654) [32], Karayaka sheep (0.028-0.044) [33], and higher than Dorper sheep (0.0011) [34]. Nucleotide and HD of mtDNA are two important indices for assessing population polymorphism and genetic differentiation; the higher the HD, the higher the genetic diversity will be, and vice versa. Since the HD and nucleotide diversity of mtDNA are high, the population polymorphism is also high [26,35]. The difference in the results might be due to the research methods, including the analysis methods, sample location, and the number of samples [36,37]. High genetic diversity in these results might be influenced by increased mutation rates in the mtDNA D-loop, the maternal effect of multiple wild ancestors, crossbreeding, and intercrossing in agricultural practices, the mixing of populations from different geographical locations, overlapping generations, natural selection, and genetic drift [36,38,39].

Genetic distance is the estimate of the closeness of genetic relationships or phylogenetics between the population, according to the Nei [30]. Genetic distance is close or equal to 0 (null) and is a very close genetic relationship. However, if the values are one or more apart, a very distant genetic relationship exists. Genetic distance values within groups of local sheep breeds on Java Island were between 0.001 and 0.040, where the value is low. This means that within the same breed, they have a close relationship. The genetic distance between groups of local sheep breeds on Java Island is around 0.004-0.036. The lowest genetic distance is between JFT and BTR or WSB (0.004), whereas the highest is between JTT and PRG (0.036). The JFT, BTR, and WSB populations have a low genetic distance value but are quite high with GRT and PRG. This means that there is a considerable genetic relationship between JFT, BTR, and WSB with GRT and PRG. These results differ from the research Prayitno *et al*. [38], who found by PCR-RAPD that BTR was closer to GRT than to JTT and JFT. However, based on the values of genetic distance according to Nei [30], the values of genetic distances in this study are still included in the category of a close genetic relationship. Need more research to find out more detail genetic diversity and genetic distances within and between populations. Further research can be carried out by comparing samples from other regions in Indonesia and from neighboring countries [40]. Apart from studying part of mtDNA, more detailed studies can also be carried out such as microsatellite [41,42], complete mitochondrial genome [27,43], whole-genome DNA [44,45], and the other studies using the next-generation sequencing [46] and/or the other methods.

### Haplotype analysis

An HD value in the range ≥0 <0.5 was included in the low category, whereas an HD value in the range >0.5 ≤1 was included in the high category [26,35]. Based on the analysis results, as presented in Table-2, the HD value of six local sheep breeds on Java Island in this study is included in the high category. The HD value and the average number of pairwise differences (K) within all tested breeds were 0.99567 and 25.36364, respectively. The HD value in this study is almost the same as fat-rumped sheep in Ethiopia (0.99) [47], five Egyptian sheep breeds (0.968) [37], four Polish sheep breeds (0.9720) [39], Savak Akkaraman sheep (0.995) [48], Iranian indigenous sheep (0.996) [31], Karayaka sheep (0.998) [33], and Mongolian native sheep (0.93) [3]. The K value in this study is similar to 10 Iranian indigenous sheep breeds (25.23) [31], but higher than five Egyptian sheep breeds (17.14782) [37], Romanian Racka sheep (6.70488) [49], and Savak Akkaraman sheep (7.068) [48].

The results of analyzing 22 individual samples were used as the basis to form 21 unique haplotypes and one shared haplotype. A comparison of the number of individual samples and the number of haplotypes (Table-2) indicated that each breed had its haplotypes that could be due to the high diversity in local sheep breeds on Java Island and/or sampling strategy (collecting unrelated individuals) [31]. This is in line with the previous studies which states that several sheep breeds in Indonesia have high genetic diversity within [39] and between [50] populations. Unique patterns in the distribution of haplotypes could be attributed to the total reproductive isolation because of the harsh geographical structure of and unique husbandry practice on Java Island, that allow farmers to select animals with unique phenotypic characters associated with the adaptability to different ecological conditions [51,52].

### Phylogenetic relationship and the maternal origin of local sheep breeds on Java Island

Domestic sheep were divided into five Hap, namely, A, B, C, D, and E [53]. Based on phylogenetic relationship analysis, this study shows that six local sheep breeds on Java Island are divided into two Hap, namely, Hap A and B (Figure-3 and Table-2). This is in line with the statement of Meadows *et al*. [53] that Indonesian sheep have two Hap, namely, Hap A and B. Hap A is mainly represented in Asian breeds, whereas Hap B is found in high frequencies in European breeds [54,55]. This study is similar to observations on four Polish sheep breeds [39], eastern Ethiopia sheep [47], and Kail sheep [56] that form two Hap, namely, Hap A and B. All JFT, JTT, BTR, and WSB sheep are classified as Hap B. Most GRT and PRG sheep are classified as Hap A, but there are GRT sheep (GRT1 and GRT2) and PRG1 sheep that are classified as Hap B. This pattern is similar to that observed for the five Egyptian sheep breeds [37], Romanian Racha Sheep [49], and Iberian sheep [57] where Hap B is predominant over Hap A. However, this in contrast to the observations of the four Nepal indigenous sheep breeds [51], Tibetan sheep [26], and native Mongolian sheep [3], where Hap A is predominant over Hap B.

This study indicates that most local sheep breeds on Java Island are descendants of European type sheep. This is also supported by the phylogenetic analysis that showed a close relationship with *Ovis musimon* (European Mouflon) (Figure-3). In contrast, Dudu *et al*. [49] stated that different breeds from Asia (Indonesia, India, and Mongolia) presented exclusively type A haplotypes. The JTT sheep in this study were all classified as Hap B. However, the study of Meadows *et al*. [53] found that the JTT sheep are divided into two Hap, namely, Hap A and B. The existence of two divisions of the two Hap is found in GRT and PRG sheep. The GRT and PRG sheep are mostly classified into Hap A, are different from other sheep breeds classified into Hap B. It could be because it is still an Asian breed (Asian Mouflon), or it can also be related to the Merino breed. Another possibility is that the GRT and PRG sheep were previously categorized as local subpopulations or strains of JTT [10]. In contrast, the two breeds were only designated as local Indonesian sheep breeds in 2011 [58] and 2017 [59]. The designation of local sheep breeds by the Ministry of Agriculture of the Republic of Indonesia and the results of this study can be the basis for future research related to the specific determination of samples of sheep breeds used. There is a need for further studies with more samples to determine the type of Hap in more detail.

According to the Ministry of Agriculture [59], the origin of the PRG sheep began in 1864 by the Dutch government who imported Merino sheep into the PRG Residency, West Java. PRG sheep are a cross between local sheep, Texel, and Merino [6]. GRT sheep are the result of crossing Merino sheep from Australia, Kaapstad sheep from Africa, and JFT sheep [60]. The phylogenetic analysis (Figure-2) shows the closeness of the PRG sheep and GRT sheep with Merino sheep from Australia (HM236174). Previous research stated that sheep from Indonesia, Mongolia, and Tibet had a close Hap A sequence [53,61]. Observations in European Mouflon have found Hap B that predominates in European breeds and minorities in Eastern Asia breeds [17,55,62,63]. However, notable exceptions are the Indonesian breeds, which may have resulted from crossbreeding with breeds of European origin [53,63].

Indonesia is an archipelago country between two continents (Asia and Australia) and two oceans (Indian and Pacific). In the past, this area was named *Nusantara*, an area with many tribes and kingdoms that were a strategic region for trade with a variety of natural resources, especially spices. Many traders from Asia, including China and Gujarat (India), and from Europe, including Britain, Portugal, and the Netherlands/Dutch, traded in this region. Java Island is one of the islands in the *Nusantara* (Indonesia) that is a strategic destination of various regions for exploring and trading [64,65]. This vantage point enables the introduction of livestock from various countries into Indonesia for development before this country became independent in 1945. It had been occupied by the Dutch government for more than 300 years [65]. The Dutch government, at this time, often brought various livestock from outside the area to be raised in this area, such as cattle, goats, pigs, horses, and sheep [60]. No study has stated that native sheep or wild sheep originated from the original geographical area of Indonesia. It is possible that the sheep developed in Indonesia currently are from sheep breeds that were introduced and developed either by the traders’ community or introduced by the Dutch government at the time.

This study shows that most local sheep breeds on Java Island are included in Hap B, which are sheep that have a close relationship with European sheep, even though this country is on the Asian continent. However, there are also local sheep breeds on Java Island that are included in Hap A. This indicates that the sheep that developed on Java Island were the result of crossing Asian and European sheep. However, this study is still limited to a small number of samples, but it can be used as a database for further research. Observations with data from mtDNA are used to determine the maternal inheritance line. It is necessary to have additional observations of the male’s line so that both maternal and paternal lines can be known to find out more about the origin of developing breeds. In Indonesia, aside from Java Island, there are still other local sheep breeds that have been established by the Ministry of Agriculture, whereas others have not despite their long development in the region. Thus, further research on these breeds still needs to be done. In-depth studies need to be performed on the genetic profiles of local Indonesian sheep breeds with more samples, a wider area, more comparative data, and various other methods that can describe in more detail the origins, characteristics, and productivity of Indonesian sheep breeds.

## Conclusion

The high genetic diversity of six local sheep breeds on Java Island suggests that they originated from different genetic resources. The JTT sheep have a closer genetic relationship to the JFT, BTR, and WSB sheep, and they are close to European sheep. In contrast, the GRT sheep have a closer genetic relationship to the PRG sheep. Both are closer to Asian sheep than they are to European sheep.

## Authors’ Contributions

WTA supervised the present study, and IGSB and RW designed and coordinated the study. AI performed the experiment, analyzed the data, and wrote the manuscript. The final manuscript has been read and developed in consultation with all authors. All authors read and approved the final manuscript.

## References

[1] Budisatria I.G.S, Panjono P, Maharani D, Ibrahim A (2018). Kambing Peranakan Etawah:Kepala Hitam atau Cokelat?.

[2] Ibrahim A, Budisatria I.G.S, Widayanti R, Artama W.T (2019). The impact of religious festival on roadside livestock traders in urban and peri-urban areas of Yogyakarta, Indonesia. Vet. World.

[3] Ganbold O, Lee S.H, Seo D, Paek W.K, Manjula P (2019). Genetic diversity and the origin of Mongolian native sheep. Livest. Sci.

[4] Cai D, Zhang N, Shao X, Sun W, Zhu S, Yang D.Y (2018). New ancient DNA data on the origins and spread of sheep and cattle in Northern China around 4000 BP. Asian Archaeol.

[5] Budisatria I.G.S, Yulianto M.D.E, Ibrahim A, Atmoko B.A, Faqar D, Profil Pedagang Ruminansia Kecil pada Periode Idul Adha di Daerah Istimewa Yogyakarta, Indonesia. Seminar Nasional Peternakan Tropis Berkelanjutan 3, Surakarta (2019).

[6] Tawaf R, Heriyadi D, Anang A, Sulaeman M, Hidayat R, Empowerment of Small Holder Farmers Business Garut Sheep in West Java (2011).

[7] Ibrahim A, Artama W.T, Widayanti R, Yulianto M.D.E, Faqar D, Budisatria I.G.S (2019). Sheep traders preferences on marketing place and their satisfaction during Eid Al-Adha period in Yogyakarta, Indonesia. IOP Conf. Ser. Earth Environ. Sci.

[8] Ibrahim A, Budisatria I.G.S, Widayanti R, Artama W.T (2019). Consumer's preferences for sheep attributes for Eid Al-Adha celebration in Yogyakarta, Indonesia. IOP Conf. Ser. Earth Environ. Sci.

[9] Ministry of Agriculture (2011). Peraturan Menteri Pertanian Nomor 48/Permentan/OT.140/9/2011 Tentang Pewilayahan Sumber Bibit.

[10] Sodiq A, Tawfik E.S (2004). Productivity and breeding strategies of sheep in Indonesia:A review. J. Agric. Rural Dev. Trop. Subtrop.

[11] Ibrahim A, Budisatria I.G.S, Widayanti R, Atmoko B.A, Yuniawan R, Artama W.T (2020). On-farm body measurements and evaluation of batur sheep on different age and sex in Banjarnegara regency, Indonesia. Adv. Anim. Vet. Sci.

[12] Wang X, Ma Y.H, Chen H, Guan W.J (2007). Genetic and phylogenetic studies of Chinese native sheep breeds (*Ovis aries*) based on mtDNA D-loop sequences. Small Rumin. Res.

[13] Bruford M.W, Bradley D.G, Luikart G (2003). DNA markers reveal the complexity of livestock domestication. Nat. Rev. Genet.

[14] Ganbold O, Lee S.H, Paek W.K, Munkhbayar M, Seo D, Manjula P, Khujuu T, Purevee E, Lee J.H (2020). Mitochondrial DNA variation and phylogeography of native Mongolian goats. Asian Aust. J. Anim. Sci.

[15] Ju Y, Liu H, He J, Wang L, Xu J, Liu H, Dong Y (2020). Genetic diversity of Aoluguya reindeer based on D-loop region of mtDNA and its conservation implications. Gene.

[16] Hiendleder S, Lewalski H, Wassmuth R, Janke A (1998). The complete mitochondrial DNA sequence of the domestic sheep (*Ovis aries*) and comparison with the other major ovine haplotype. J. Mol. Evol.

[17] Hiendleder S, Kaupe B, Wassmuth R, Janke A (2002). Molecular analysis of wild and domestic sheep questions current nomenclature and provides evidence for domestication from two different subspecies. Proc. R. Soc. B Biol. Sci.

[18] Kõressaar T, Lepamets M, Kaplinski L, Raime K, Andreson R, Remm M (2018). Primer3-masker:Integrating masking of template sequence with primer design software. Bioinformatics.

[19] Kumar S, Stecher G, Tamura K (2016). MEGA7:Molecular evolutionary genetics analysis version 7.0 for bigger datasets. Mol. Biol. Evol.

[20] Thompson J.D, Higgins D.G, Gibson T.J (1994). CLUSTAL W:Improving the sensitivity of progressive multiple sequence alignment through sequence weighting, position-specific gap penalties and weight matrix choice. Nucleic Acids Res.

[21] Kimura M (1980). A simple method for estimating evolutionary rates of base substitutions through comparative studies of nucleotide sequences. J. Mol. Evol.

[22] Rozas J, Ferrer-Mata A, Sanchez-DelBarrio J.C, Guirao-Rico S, Librado P, Ramos-Onsins S.E, Sanchez-Gracia A (2017). DnaSP v6:DNA sequence polymorphism analysis of large datasets. Mol. Biol. Evol.

[23] Meadows J.R.S, Hiendleder S, Kijas J.W (2011). Haplogroup relationships between domestic and wild sheep resolved using a mitogenome panel. Heredity (Edinb).

[24] Saitou N, Nei M (1987). The Neighbor-joining method :A new method for reconstructing phylogenetic trees. Mol. Biol. Evol.

[25] Felsenstein J (1985). Confidence limits on phylogenies:An approach using the bootstrap. Evolution (N.Y).

[26] Liu J, Ding X, Zeng Y, Yue Y, Guo X, Guo T, Chu M, Wang F, Han J, Feng R, Sun X, Niu C, Yang B, Guo J, Yuan C (2016). Genetic diversity and phylogenetic evolution of Tibetan sheep based on mtDNA D-loop sequences. PLoS One.

[27] Liu J.B, Ding X.Z, Guo T.T, Yue Y.J, Zeng Y.F, Guo X, Chu M, Han J.L, Sun X.P, Niu C.E, Yang B.H, Guo J, Yuan C (2016). The complete mitochondrial genome sequence of the wild Huoba Tibetan sheep of the Qinghai-Tibetan Plateau in China. Mitochondrial DNA Part A.

[28] Fan H, Zhao F, Zhu C, Li F, Liu J, Zhang L, Wei C, Du L (2016). Complete mitochondrial genome sequences of Chinese indigenous sheep with different tail types and an analysis of phylogenetic evolution in domestic sheep. Asian Aust. J. Anim. Sci.

[29] Qiao G, Zhang H, Zhu S, Yuan C, Zhao H, Han M, Yue Y, Yang B (2020). The complete mitochondrial genome sequence and phylogenetic analysis of Alpine Merino sheep (*Ovis aries*). Mitochondrial DNA Part B.

[30] Nei M (1987). Molecular Evolutionary Genetics.

[31] Rafia P, Tarang A (2016). Sequence variations of mitochondrial DNA displacement-loop in Iranian Indigenous sheep breeds. Iran. J. Appl. Anim. Sci.

[32] Othman O.E.M, Payet-Duprat N, Harkat S, Laoun A, Maftah A, Lafri M, Da Silva A (2016). Sheep diversity of five Egyptian breeds:Genetic proximity revealed between desert breeds:Local sheep breeds diversity in Egypt. Small Rumin. Res.

[33] Kirikci K, Noce A, Akif M, Mercan L, Amills M, Cam M.A, Mercan L, Amills M (2018). The analysis of mitochondrial data indicates the existence of population substructure in Karayaka sheep. Small Rumin. Res.

[34] de Oliveira J.A, do Egito A.A, do Amaral Crispim B, de Vargas Junior F.M, de Oliveira Seno L, Barufatti A (2020). Importance of naturalized breeds as a base for the formation of exotic sheep (*Ovis aries*) breeds in tropical altitude regions. Genet. Mol. Biol.

[35] Zhao Y, Zhao E, Zhang N, Duan C (2011). Mitochondrial DNA diversity, origin, and phylogenic relationships of three Chinese large-fat-tailed sheep breeds. Trop. Anim. Health Prod.

[36] Selvam R, Murali N, Thiruvenkadan A.K, Saravanakumar R, Ponnudurai G, Jawahar T.P (2017). Single-nucleotide polymorphism-based genetic diversity analysis of the Kilakarsal and Vembur sheep breeds. Vet. World.

[37] Othman O.E, Balabel E.A, Abdel-Samad M.F (2014). Mitochondrial DNA diversity in five Egyptian sheep breeds. Glob. Vet.

[38] Prayitno P, Hartatik T, Pratiwi R, Artama W.T (2008). Genetic relatedness between Batur, Merino and local sheep based on random amplified polymorphism DNA marker. Anim. Prod.

[39] Koseniuk A, Słota E (2016). Mitochondrial control region diversity in Polish sheep breeds. Arch. Anim. Breed.

[40] Kim Y.S, Tseveen K, Batsukh B, Seong J, Kong H.S (2020). Origin-related study of genetic diversity and heteroplasmy of Mongolian sheep (*Ovis aries*) using mitochondrial DNA. J. Anim. Reprod. Biotechnol.

[41] Amareswari P, Gnana Prakash M, Ekambaram B, Mahendar M, Krishna C.H (2018). Molecular genetic studies on Nellore and Deccani sheep using microsatellite markers. Indian J. Anim. Res.

[42] Abdelkader A.A, Ata N, Benyoucef M.T, Djaout A, Azzi N, Yilmaz O, Cemal, İ, Gaouar S.B.S (2018). New genetic identification and characterisation of 12 Algerian sheep breeds by microsatellite markers. Ital. J. Anim. Sci.

[43] Wang C, Xu H, Li D, Wu J, Wen A, Xie M, Wang Q, Zhu G, Ni Q, Zhang M, Yao Y (2020). Phylogenetic and characterization of the complete mitochondrial genome relationship of Argali sheep (*Ovis ammon*). Mitochondrial DNA Part B Resour.

[44] Heaton M.P, Smith T.P.L, Freking B.A, Workman A.M, Bennett G.L, Carnahan J.K, Kalbfleisch T.S (2017). Using sheep genomes from diverse U.S. Breeds to identify missense variants in genes affecting fecundity. F1000 Research.

[45] Deniskova T.E, Dotsev A.V, Selionova M.I, Kunz E, Medugorac I, Reyer H, Wimmers K, Barbato M, Traspov A.A, Brem G, Zinovieva N.A (2018). Population structure and genetic diversity of 25 Russian sheep breeds based on whole-genome genotyping. Genet. Sel. Evol.

[46] Dunisławska A, Łachmańska J, Sławińska A, Siwek M (2017). Next generation sequencing in animal science-a review. Anim. Sci. Pap. Reports.

[47] Nigussie H, Mwacharo J.M, Osama S, Agaba M, Mekasha Y, Kebede K, Abegaz S, Pal S.K (2019). Genetic diversity and matrilineal genetic origin of fat-rumped sheep in Ethiopia. Trop. Anim. Health Prod.

[48] Yağci S, Baş S, Kiraz S (2020). Study of mitochondrial DNA (mtDNA) D-loop region polymorphism in Şavak Akkaraman sheep. Turk. J. Vet. Anim. Sci.

[49] Dudu A, Ghita E, Costache M, Georgescu S.E (2016). Origin and genetic diversity of Romanian Racka sheep using mitochondrial markers. Small Rumin. Res.

[50] Jakaria J, Zein M.S.A, Sulandari S, Subandriyo S, Muladno M (2012). The use of microsatellite markers to study genetic diversity in Indonesia sheep. J. Indones. Trop. Anim. Agric.

[51] Gorkhali N.A, Han J.L, Ma Y.H (2015). Mitochondrial DNA variation in indigenous sheep (*Ovis aries*) breeds of Nepal. Trop. Agric. Res.

[52] Yang J, Li W.R, Lv F.H, He S.G, Tian S.L, Peng W.F, Sun Y.W, Zhao Y.X, Tu X.L, Zhang M, Xie X.L, Wang Y.T, Li J.Q, Liu Y.G, Shen Z.Q, Wang F, Liu G.J, Lu H.F, Kantanen J, Han J.L, Li M.H, Liu M.J (2016). Whole-genome sequencing of native sheep provides insights into rapid adaptations to extreme environments. Mol. Biol. Evol.

[53] Meadows J.R.S, Li K, Kantanen J, Tapio M, Sipos W, Pardeshi V, Gupta V, Calvo J.H, Whan V, Norris B, Kijas J.W (2005). Mitochondrial sequence reveals high levels of gene flow between breeds of domestic sheep from Asia and Europe. J. Hered.

[54] Hiendleder S, Mainz K, Plante Y, Lewalski H (1998). Analysis of mitochondrial DNA indicates that domestic sheep are derived from two different ancestral maternal sources:No evidence for contributions from urial and argali sheep. J. Hered.

[55] Mereu P, Pirastru M, Barbato M, Satta V, Hadjisterkotis E, Manca L, Naitana S, Leoni G.G (2019). Identification of an ancestral haplotype in the mitochondrial phylogeny of the ovine haplogroup B. Peer J.

[56] Hussain T, Babar M.E, Wajid A (2016). Extra nuclear DNA control region and *cytochrome b* gene based phylogeny of kail sheep breed of Azad Jammu and Kashmir:Implications towards conservation. J. Anim. Plant Sci.

[57] Pedrosa S, Arranz J.J, Brito N, Renseigné N, San Primitivo F, Bayón Y (2007). Mitochondrial diversity and the origin of Iberian sheep. Genet. Sel. Evol.

[58] Ministry of Agriculture (2011). Keputusan Menteri Pertanian Nomor 2914/Kpts/OT.140/6/2011 Tentang Penetapan Rumpun Domba Garut.

[59] Ministry of Agriculture (2017). Keputusan Menteri Pertanian Republik Indonesia Nomor 300/Kpts/SR.120/5/2017 Tentang Penetapan Rumpun Domba Priangan.

[60] Directorate General of Livestock Services (2003). National Report on Animal Genetic Resources Indonesia, A Strategic Policy Document.

[61] Guangxin E, Yong-Ju Z, Ri-Su N, Yue-Hui M, Jia-Hua Z, Li-Peng C, Xiao-Yu Q, Zhong-Quan Z, Ya-Wang S, Xin W, Yong-Fu H (2017). Meta-analysis evidence of maternal lineages in Chinese Tibetan sheep using mtDNA D-loop panel. Mitochondrial DNA A DNA Mapp. Seq. Anal.

[62] Guo J, Du L.X.L, Ma Y.H, Guan W.J, Li H.B, Zhao Q.J, Li X, Rao A.Q, Rao S.Q (2005). A novel maternal lineage revealed in sheep (*Ovis aries*). Anim. Genet.

[63] Tapio M, Marzanov N, Ozerov M, Ćinkulov M, Gonzarenko G, Kiselyova T, Murawski M, Viinalass H, Kantanen J (2006). Sheep mitochondrial DNA variation in European, Caucasian, and Central Asian areas. Mol. Biol. Evol.

[64] Ricklefs M.C (1981). A History of Modern Indonesia c. 1300 to the Present.

[65] Vickers A (2005). A History of Modern Indonesia.

